# Age or age of onset: which is the best criterion to classify late-life depression?

**DOI:** 10.1097/YIC.0000000000000472

**Published:** 2023-03-21

**Authors:** Paolo Olgiati, Giuseppe Fanelli, Alessandro Serretti

**Affiliations:** a Department of Biomedical and Neuromotor Sciences, University of Bologna, Italy; b Department of Human Genetics, Radboud University Medical Center, Donders Institute for Brain, Cognition and Behaviour, Nijmegen, The Netherlands

**Keywords:** age of onsetantidepressant treatment, antidepressant response, brain ageing, cognitive impairment, diagnostic criteria, geriatric depression, geriatric psychiatry, major depression, old age, mood disorders, neuropsychological testing

## Abstract

In late-life depression (LLD), several differences between patients whose first episode is reported after age 65 (late-onset depression, LOD) and those with early-onset depression (EOD) might reflect the effects of brain ageing. To test this hypothesis, we analysed the impact of current age and age at illness onset on a number of clinical and cognitive manifestations in 438 outpatients with major depressive disorder aged >60 years, treated with venlafaxine for 12 weeks. When compared to the EOD group, patients with LOD were older (*P* < 0.00001) and associated with lower depression severity (*P* = 0.0029), lower global cognitive functioning [Mini-Mental State Examination (MMSE): *P* = 0.0001; Repeatable Battery for the Assessment of Neuropsychological Status: immediate memory, *P* = 0.0009, and delayed memory, *P* < 0.00001; Delis-Kaplan Executive Function System measuring executive functions: Trail-Making Test (TMT) – *P* = 0.0004 and Colour-Word Interference Test, Inhibition – *P* = 0.0063], and more dyskinesias (Abnormal Involuntary Movement Scale: *P* = 0.0006). After controlling for its interactions with age of onset, current age was inversely correlated with Montgomery Åsberg Depression Rating Scale scores at baseline (*P* < 0.00001) and week 12 (*P* = 0.0066), MMSE (*P* < 0.00001), delayed memory (*P* < 0.00001), and TMT (*P* = 0.0021). Age of onset predicted impairment in immediate (*P* = 0.023) and delayed memory (*P* = 0.0181), and dyskinesias (*P* = 0.0006). Although most features of LLD are related to ageing rather than to late-onset, LOD is a possible separate diagnostic entity characterised by memory dysfunction and increased liability to movement disorders.

## Introduction

Depression is a common and serious health concern in older people. In this group, the prevalence of any depressive disorder ranges from less than 10% to over 40% depending on diagnostic thresholds and settings (Beekman *et al*., 1999; Djernes, 2006). The occurrence of a depressive disorder after the age of 60 is called late-life depression (LLD). At the clinical level, LLD has been characterised by chronic course (Comijs *et al*., 2015) and increased morbidity and mortality (Unützer, 2007; Wei *et al.*, 2022). At the diagnostic level, LLD is a broad term that does not differentiate between patients who are depressed for the first time in late life (late-onset depression, LOD) and those who experience late-life relapses of depressive disorders already present during their youth or adulthood (early-onset depression, EOD). In psychogeriatrics, a large amount of literature has shown that LOD and EOD are associated with distinct clinical (Holroyd and Duryee, 1997; Benazzi, 2004; Xiao *et al.*, 2020), neurocognitive (Sachs-Ericsson *et al.*, 2013; Mackin *et al.*, 2014), and morphological (Ballmaier *et al.*, 2008) features. In addition, several potential peripheral biomarkers for depression in the elderly, possibly related to the specific symptom profile and disease phase, have been suggested (Vogelzangs et al., 2014; Fanelli et al., 2019, 2020). Recent studies have also reported differences between early and late-onset suicide attempters (Kenneally *et al*., 2019; Szücs *et al*., 2020; Perry *et al*., 2021). All these findings apparently support age at onset of the first depressive episode as a stem diagnostic criterion for LLD. On the other hand, ageing process is itself characterised by physiological changes that involve the central nervous system, with consequences for neurological and cognitive functions (Amarya *et al*., 2018). In the geriatric population increasing age has been associated with greater cognitive impairment, loss of autonomy and diminished quality of life (Cohen-Mansfield *et al.*, 2013; Kim, 2021). Such age-related differences could be reproduced in LLD. Thus, although most findings point to a clear-cut distinction between LLD and adult depression, their interpretation is actually a conundrum because some of them could be specific characteristics of LOD, whereas others could simply mirror the effects of ageing. However, this bias is seldom considered in LLD research.

The general purpose of this study was to disentangle the role of current age and age of onset as classifiers for LLD. In detail, we used data from a large sample of depressed outpatients aged 60 years or older with the following goals: (1) to test the association of current age and age of onset with a number of clinical and neurocognitive variables related to LLD severity; (2) to investigate the impact of age and age of onset on treatment adherence and tolerability; and (3) to analyse interactions between current age and age of onset as moderators of LLD.

## Methods

### Study sample

We performed a secondary analysis of the IRL GREY (Incomplete Response in Late-Life Depression: Getting to Remission) sample (NCT00892047). IRL GREY is a multi-site study including subjects aged 60 and older, with a Diagnostic and Statistical Manual of mental disorders (DSM)-IV diagnosis of major depressive disorder (confirmed by the Structured Clinical Interview for DSM-IV Axis I Disorders (SCID-I) and a Montgomery Åsberg Depression Rating Scale (MADRS) score of at least 15). Exclusion criteria were dementia [based upon DSM-IV criteria, as well as a Mini-Mental State Examination (MMSE) score less than 24], lifetime diagnoses of bipolar or psychotic disorders, alcohol or substance abuse/dependence within the past 3 months and high risk for suicide (e.g. current/recent intent or plan) (Joel *et al.*, 2014).

All subjects were included in the IRL GREY trial after obtaining their written informed consent. This research group certifies that data collected from the IRL GREY trial were exclusively used for scientific investigation and, before obtaining access to them, the objectives of our investigation were clearly reported in a request form.

### Treatment and assessment

The IRL GREY trial design included three treatment phases, each lasting 12 weeks. During phase 1, eligible patients who gave their written informed consent were openly treated with venlafaxine XR (up to 300 mg/day). At the end of this phase, participants with incomplete response were randomised to receive either aripiprazole (2–15 mg/day; target dose: 10 mg/day) or placebo in addition to venlafaxine (phase 2), with the goal of achieving remission (two consecutive assessments with a ≤10 MADRS score). Finally, phase 3 patients who had remitted were maintained in the treatment arm to which they had been assigned during phase 2, in order to determine the stability of remission (Joel *et al.*, 2014). Our analysis was based on data collected during phase 1.

The psychopathological assessment included the SCID-I (First et al., 1996), the MADRS (Montgomery and Åsberg, 1979), and the 17-item Hamilton Depression Rating Scale (HDRS_17_) (Hamilton, 1960) for depressive symptomatology, the Brief Symptom Inventory (BSI: six-item self-report questionnaire rated on a 0–5 scale) (Derogatis and Melisaratos, 1983), Anxiety Sensitivity Index (ASI; fear of panic; 16 items; 0–4 score range) (Reiss *et al.*, 1986), and Penn State Worry Questionnaire (PSWQ; 16 items; 1–5 score range) (Meyer *et al.*, 1990) to investigate anxiety-related manifestations and Suicide Ideation Scale (SIS; 21 items) (Beck *et al*., 1979).

Cognitive assessment was performed including the MMSE (Folstein *et al*., 1975) for global cognitive functioning, the Repeatable Battery for the Assessment of Neuropsychological Status (RBANS) (Randolph *et al.*, 1998) to evaluate memory performance, and two tests [i.e. the Colour-Word Interference Test (CWIT) – Inhibition part, and Trail-Making Test (TMT)] included in the Delis-Kaplan Executive Function System (D-KEFS), which is a neuropsychological test battery evaluating executive functioning (Fine and Delis, 2011). Health assessment was completed by the Cumulative Illness Rating Scale for Geriatrics (CIRS-G) (Linn *et al*., 1968; Miller *et al.*, 1992), which rates 13 organ systems from 0 (no problem) to 4 (organ failure) and the 36-item Short Form Health Survey (SF-36) (Ware and Sherbourne, 1992) as a measure of health-related quality of life whereas side effects due to antidepressant treatment were ascertained using the Udvalg for Klinske Undersogelser Side Effects Scale (Lingjaerde *et al.*, 1987), Abnormal Involuntary Movement Scale (AIMS) (Lane *et al.*, 1985) and Barnes Akathisia Scale (Barnes, 1989).

### Statistical analyses

The association of current age and age of onset with clinical variables, such as baseline depression score, suicidal ideation, antidepressant response, and cognitive domains, was explored in the IRL GREY sample. After a preliminary power analysis conducted using G*Power 3 (Faul *et al*., 2007), age (young–old: 60–74 years vs. old–old: ≥75 years) and age of onset (EOD: <65 years vs. LOD: ≥65 years) groups were compared by means of univariate tests (Student’s *t* or χ^2^ tests as appropriate).

The statistical significance threshold was set at *α* = 0.025 due to the number of comparisons, but without a formal correction for multiple testing given the *a priori* hypothesis (Amrhein *et al*., 2019).

In subsequent steps, age and age of onset were included in multiple regression or logistic regression models to ascertain their interacting effects as predictors of clinical variables.

Statistical analyses were conducted in OpenStat version 8 December 2014 (https://openstat.info/OpenStatMain.htm).

## Results

### Sample characteristics and power analysis

Our secondary analysis involved 438 patients with moderate to severe depression (males: 151; non-Caucasian: 51) whose clinical features are summarised as follows: MADRS: 26.59 ± 5.67; suicidal ideation: 2.37 ± 4.51; cognitive function: RBANS (total memory): 95.31 ± 15.90; D-KEFS measuring executive functions: CWIT (response inhibition and cognitive flexibility): 8.75 ± 4.05; TMT (attention, cognitive flexibility, and working memory): 10.30 ± 3.02. Of the 438 patients, 347 (80%) were aged between 60 and 74 years, and 91 (20%) were ≥75 years old. Age of onset of the first depressive episode was 42.04 ± 21.67 years, with 76 patients who became depressed after the age of 65 (LOD) and 362 earlier (EOD).

The results of the IRL GREY trial’s primary analyses have been reported elsewhere (Joel *et al*., 2014; Lenze *et al.*, 2015).

Preliminary power analysis suggested that our sample was enough powered (0.80) to detect small-medium effect sizes (*d* = 0.36) in comparisons between 60–74 and ≥75 years groups, corresponding to differences of 2 points for MADRS, 1.6 points for suicidal ideation, 6 points for memory tasks, and 1.3 points for executive function tests. As regards age of onset groups, the minimum detectable effect size was *d* = 0.42 which corresponded to differences of 2.3 points for MADRS, 1.9 points for suicidal ideation, 7 points for memory tasks, and 1.5 points for executive function domains.

### Comparisons between age groups

In comparison with the 60–74 years old counterpart, subjects aged ≥75 years were characterised by later depression onset (58.30 vs. 37.78 years, *P* < 0.00001), less severe symptomatology (MADRS) reported at baseline (24.29 vs. 27.20, *P* < 0.00001) and after 12 treatment weeks (8.83 vs. 14.78, *P* < 0.00001), more somatic comorbidities (CIRS-G: 10.89 vs. 9.68, *P* = 0.0212), and drop-out cases (27/91 vs. 58/347, *P* = 0.0050). Although older subjects received lower dosages of venlafaxine compared to the younger group (193.35 mg vs. 234.89 mg, *P* < 0.0001), they had more dyskinesias (AIMS: 0.30 vs. 0.17, *P* = 0.0198). In terms of cognitive functioning ≥75 years old group was associated with reduced MMSE scores (28.04 vs. 28.88, *P* < 0.00001) and worse performances in memory (RBANS: immediate memory: 92.55 vs. 98.55, *P* = 0.0110; delayed memory: 89.61 vs. 98.40, *P* < 0.00001) and executive function tests (D-KEFS: TMT – 7.01 vs. 9.18, *P* < 0.00001; CWIT Inhibition – 9.11 vs. 10.25, *P* = 0.0107) (see Table 1).

**Table 1:** Late-life depression: comparison between the ≥75 years old and 60–74 years old groups

	≥75 years (*N* = 91)	60–74 years (*N* = 347)	*P* value
Demographic characteristics			
Age	80.47 ± 4.64	66.13 ± 4.18	<0.0001*
Males, *n* (%)	27 (0.29)	124 (0.36)	0.2792
Caucasians, *n* (%)	83 (0.91)	304 (0.87)	0.1703
Education years	14.27 ± 3.12	14.50 ± 2.77	0.4990
Mental health			
MADRS baseline	24.29 ± 5.51	27.20 ± 5.56	<0.0001*
MADRS week 12	8.83 ± 7.85	14.78 ± 10.70	<0.0001*
Suicide ideation (SIS)	1.77 ± 3.96	2.52 ± 4.64	0.1578
ASI (anxiety)	25.15 ± 12.74	25.66 ± 12.73	0.7378
BSI (anxiety)	1.76 ± 1.01	1.89 ± 0.95	0.2403
PSWQ (anxiety)	58.62 ± 11.75	59.85 ± 13.58	0.4393
GAD (SCID-I), *n* (%)	18 (0.20)	72 (0.21)	0.7684
Panic disorder (SCID-I), *n* (%)	7 (0.08)	32 (0.09)	0.6104
OCD (SCID-I), *n* (%)	3 (0.03)	11 (0.03)	0.9999
Social phobia (SCID-I), *n* (%)	4 (0.04)	32 (0.09)	0.1243
Late-onset insomnia	0.99 ± 0.89	1.07 ± 0.90	0.4739
Middle insomnia	0.91 ± 0.84	1.19 ± 0.85	0.0056*
Early awakening	0.80 ± 0.85	0.86 ± 0.88	0.5435
SF-36 mental health	30.00 ± 7.39	26.60 ± 9.21	0.0018*
Cognitive functioning			
MMSE	28.01 ± 1.91	28.88 ± 1.31	<0.0001*
RBANS Attention index	97.02 ± 17.73	99.76 ± 16.63	0.1778
RBANS Language index	96.12 ± 14.31	98.77 ± 11.46	0.0713
RBANS Visuospatial index	87.89 ± 17.96	93.24 ± 16.98	0.0099*
RBANS Immediate memory	92.55 ± 18.99	99.08 ± 17.89	0.0110*
RBANS Delayed memory	89.61 ± 18.97	98.40 ± 14.05	<0.0001*
RBANS Memory total score	90.54 ± 16.76	96.51 ± 15.49	0.0019*
D-KEFS Trail-making Test	7.01 ± 4.46	9.18 ± 3.83	<0.0001*
D-KEFS CWIT	9.11 ± 4.35	10.25 ± 3.33	0.0107*
Physical health			
Somatic illness (CIRS-G)	10.89 ± 4.73	9.68 ± 4.34	0.0212*
SF-36 physical health	40.46 ± 11.30	43.22 ± 11.61	0.0473
Side effects and drop-out			
All side effects (UKU)	16.97 ± 6.37	17.72 ± 7.07	0.3589
Dyskinesias (AIMS)	0.30 ± 0.64	0.17 ± 0.42	0.0198*
Akathisia (BARS)	0.34 ± 0.56	0.47 ± 0.64	0.0715
Venlafaxine dose	193.35 ± 82.26	234.50 ± 76.79	<0.0001*
Drop-out, *n* (%)	27 (0.30)	58 (0.17)	0.0055*

The mean ± SD is shown for each variable, unless otherwise specified.

*Statistically significant (*P* < 0.025).

ASI, Anxiety Sensitivity Index; AIMS, Abnormal Involuntary Movement Scale; BARS, Barnes Akathisia Rating Scale; BSI, Brief Symptom Inventory; CWIT, Colour-Word Interference Test; CIRS-G, Cumulative Illness Rating Scale for Geriatrics; D-KEFS, Delis-Kaplan Executive Function System; GAD, generalised anxiety disorder; MADRS, Montgomery Åsberg Depression Rating Scale; MMSE, Mini-Mental State Examination; *N*, sample size; OCD, obsessive-compulsive disorder; PSWQ, Penn State Worry Questionnaire; RBANS, Repeatable Battery for the Assessment of Neuropsychological Status; SIS, Suicide Ideation Scale; SCID-I, Structured Clinical Interview for DSM-IV Axis I Disorders; SF-36, 36-item Short Form Health Survey; UKU, Udvalg for Klinske Undersogelser.

### Late-onset depression vs. early-onset depression

Compared to patients with EOD, those with LOD were associated with older age (77.89 vs. 67.27, *P* < 0.00001), lower depression scores at baseline (MADRS: 24.84 vs. 26.97, *P* = 0.0029) and at week 12 (MADRS: 10.42 vs. 14.30, *P* = 0.0084), and lower anxiety levels (PSWQ: 55.82 vs. 60.34, *P* = 0.0081). Conversely, no significant differences were seen in terms of somatic comorbidity (CIRS-G: 10.58 vs. 9.80, *P* = 0.1654) and drop-out rate (19/76 vs. 66/362, *P* = 0.1750). Regarding cognitive functioning, patients with LOD were characterised by lower MMSE scores (28.06 vs. 28.85, *P* = 0.0001), greater memory impairment (immediate: 90.66 vs. 98.30, *P* = 0.0009; delayed: 89.04 vs. 98.23, *P* < 0.00001), and greater executive dysfunction (TMT: 7.21 vs. 9.07, *P* = 0.0004; CWIT Inhibition: 8.96 vs. 10.24, *P* = 0.0063). Finally, LOD subjects had higher dyskinesia levels (AIMS: 0.30 vs. 0.16, *P* = 0.0013) notwithstanding they received lower venlafaxine doses (see Table 2).

**Table 2:** Late-life depression: comparison between the late-onset depression (age of onset ≥60 years) and early-onset depression (age of onset <60) groups

	LOD (*N* = 76)	EOD (*N* = 362)	*P* value

Demographic characteristics			
Age	77.89 ± 6.87	67.27 ± 5.81	<0.0001*
Males, *n* (%)	31 (0.41)	120 (0.33)	0.2032
Caucasians, *n* (%)	67 (0.88)	320 (0.88)	0.9999
Education years	13.88 ± 2.92	14.57 ± 2.82	0.0533
Mental health			
MADRS baseline	24.84 ± 5.69	26.97 ± 5.60	0.0029*
MADRS week 12	10.42 ± 8.92	14.30 ± 10.66	0.0084*
Suicide ideation (SIS)	2.05 ± 4.43	2.43 ± 4.54	0.5060
ASI (anxiety)	25.22 ± 12.31	25.62 ± 12.81	0.8058
BSI (anxiety)	1.77 ± 1.02	1.90 ± 0.96	0.2964
PSWQ (anxiety)	55.82 ± 13.88	60.34 ± 13.10	0.0081*
GAD (SCID-I), *n* (%)	15 (0.20)	77 (0.21)	0.7656
Panic disorder (SCID-I), *n* (%)	3 (0.04)	38 (0.10)	0.0751
OCD (SCID-I), *n* (%)	4 (0.05)	10 (0.03)	0.2604
Social phobia (SCID-I), *n* (%)	4 (0.05)	34 (0.09)	0.2455
Late-onset insomnia	1.03 ± 0.94	1.05 ± 0.89	0.4739
Middle insomnia	1.07 ± 0.90	1.14 ± 0.84	0.4653
Early awakening	0.82 ± 0.89	0.86 ± 0.87	0.6612
SF-36 mental health	29.58 ± 8.34	26.75 ± 9.01	0.0143*
Cognitive functioning			
MMSE	28.06 ± 1.82	28.85 ± 1.36	0.0001*
RBANS Total score	89.56 ± 17.13	96.55 ± 15.38	0.0005*
RBANS Attention index	95.76 ± 19.51	99.93 ± 16.20	0.0540
RBANS Language index	95.78 ± 14.35	98.77 ± 11.53	0.0530
RBANS Visuospatial index	88.05 ± 18.04	93.02 ± 17.03	0.0239*
RBANS Immediate memory	90.66 ± 17.81	98.30 ± 18.06	0.0009*
RBANS Delayed memory	89.04 ± 18.78	98.23 ± 14.32	<0.0001*
D-KEFS Trail-Making Test	7.21 ± 4.48	9.07 ± 3.88	0.0005*
D-KEFS CWIT	8.96 ± 4.50	10.24 ± 3.32	0.0063*
Physical health			
Somatic illness (CIRS-G)	10.58 ± 4.68	9.80 ± 4.39	0.1654
SF-36 physical health	41.97 ± 11.40	42.80 ± 11.64	0.5814
Side-effects and drop out			
All side effects (UKU)	16.62 ± 6.57	17.76 ± 6.99	0.1924
Dyskinesias (AIMS)	0.30 ± 0.64	0.16 ± 0.41	0.0013*
Akathisia (BARS)	0.45 ± 0.66	0.45 ± 0.62	0.9861
Venlafaxine dose	198.45 ± 82.51	232.10 ± 77.92	0.0008*
Drop-out, *n* (%)	19 (0.25)	66 (0.18)	0.1755

The mean ± SD is shown for each variable, unless otherwise specified.

*Statistically significant (*P* < 0.025).

AIMS, Abnormal Involuntary Movement Scale; ASI, Anxiety Sensitivity Index; BARS, Barnes Akathisia Rating Scale; BSI, Brief Symptom Inventory; CWIT, Colour-Word Interference Test; CIRS-G, Cumulative Illness Rating Scale for Geriatrics; D-KEFS, Delis-Kaplan Executive Function System; EOD, early-onset depression; GAD, generalised anxiety disorder; LOD, late-onset depression; MADRS, Montgomery Åsberg Depression Rating Scale; MMSE, Mini-Mental State Examination; OCD, obsessive-compulsive disorder; PSWQ, Penn State Worry Questionnaire; RBANS, Repeatable Battery for the Assessment of Neuropsychological Status; SCID-I, Structured Clinical Interview for DSM-IV Axis I Disorders; SIS, Suicide Ideation Scale; SF-36, 36-item Short Form Health Survey; UKU, Udvalg for Klinske Undersogelser.

### Analysis of the age of onset x current age interactions

By controlling for age of onset, current age confirmed its positive correlations with somatic comorbidities (CIRS-G: *β* = 0.130, *P* = 0.0080; SF-36 physical health: *β* = −0.132, *P* = 0.0069) and drop-out (OR = 2.21; 95% confidence interval, 1.16–4.19) as well as its inverse correlations with depression score at baseline (*β* = −0.209, *P* < 0.00001) and after 12 weeks of treatment (*β* = −0.142, *P* = 0.0066), global cognitive functioning (MMSE; *β* = −0.206, *P* < 0.00001), delayed memory (*β* = −0.156, *P* = 0.0061), and TMT score (*β* = −0.179, *P* = 0.0021). Moreover, age was inversely related to akathisia (*β* = −0.119, *P* = 0.0386) after controlling for venlafaxine dose (Table 3). Conversely, age of onset was a predictor of memory dysfunction (RBANS; immediate memory: *β* = −0.132, *P* = 0.023; delayed memory: *β* = −0.135, *P* = 0.0181) and dyskinesias (AIMS: *β* = 0.176, *P* = 0.0006).

**Table 3:** Late-life depression: role of current age and age of onset

Dependent variable	Predicting variables	Significant predictors
MADRS (baseline)	Current age; age of onset	Current age
		*β* = −0.209, *P* < 0.0001
MADRS (week 12)	Current age; age of onset; baseline MADRS	Baseline MADRS
		*β* = 0.388, *P* < 0.0001
		Current age
		*β* = −0142, *P* = 0.0066
PSWQ	Current age; age of onset; baseline MADRS	Baseline MADRS
		*β* = 0.336, *P* < 0.0001
Middle insomnia	Current age; age of onset; baseline MADRS	Baseline MADRS
		*β* = 0.200, *P* < 0.0001
SF-36 mental health	Current age; age of onset; baseline MADRS	Baseline MADRS
		*β* = −0.475, *P* < 0.0001
SF-36 physical health	Current age; age of onset; baseline MADRS	Baseline MADRS
		*β* = −0.187, *P* = 0.0001
		Current age
		*β* = −0.132, *P* = 0.0069
Somatic illness (CIRS-G)	Current age; age of onset; baseline MADRS	Current age
		*β* = −0.130, *P* = 0.0080
MMSE	Current age; age of onset; baseline MADRS	Current age
		*β* = −0.206, *P* < 0.0001
RBANS Visuospatial index	Current age; age of onset; baseline MADRS	None
RBANS Immediate memory	Current age; age of onset; baseline MADRS	Age of onset
		*β* = −0.132, *P* = 0.0223
RBANS Delayed memory	Current age; age of onset; baseline MADRS	Current age
		*β* = −0.156, *P* = 0.0061
		Age of onset
		*β* = −0.135, *P* = 0.0181
RBANS Total memory	Current age; age of onset; baseline MADRS	Age of onset
		*β* = −0.119, *P* = 0.0428
D-KEFS Trail-Making Test	Current age; age of onset; baseline MADRS	Current age
		*β* = −0.179, *P* = 0.0021
D-KEFS CWIT	Current age; age of onset; baseline MADRS	None
Dyskinesias (AIMS)	Current age; age of onset; venlafaxine dose	Age of onset
		*β* = 0.176, *P* = 0.0006
All side effects (UKU)	Current age; age of onset; venlafaxine dose	Venlafaxine dose
		*β* = 0.109, *P* = 0.0250
Akathisia (BARS)	Current age; age of onset; venlafaxine dose	Current age
		*β* = −0.119, *P* = 0.0386
Drop-out	Current age; age of onset; AIMS; UKU; BAS	Current age
		OR 2.21 [1.16–4.19]

AIMS, Abnormal Involuntary Movement Scale; BARS, Barnes Akathisia Rating Scale; CIRS-G, Cumulative Illness Rating Scale for Geriatrics; D-KEFS, Delis-Kaplan Executive Function System; Colour-Word Interference Test; MADRS, Montgomery Åsberg Depression Rating Scale; MMSE, Mini-Mental State Examination; PSWQ, Penn State Worry Questionnaire; RBANS, Repeatable Battery for the Assessment of Neuropsychological Status; SF-36, 36-item Short Form Health Survey; UKU, Udvalg for Klinske Undersogelser.

## Discussion

This study aimed to investigate the role of current age and age at onset of first depressive episode as specifiers for late-life depressive disorder. These two parameters were found to be highly interrelated in the IRL GREY sample, therefore it was interesting to disentangle their specific effects.

Most characteristics reported in LOD did not appear to be typical manifestations of the disorder but, rather, consequences of older age. In particular, older individuals had less severe depression symptoms but more cognitive impairment than their younger counterparts. The negative correlation between age and depression severity was already known in previous studies. It had been suggested, for example, by an analysis of the National Comorbidity Survey-Replication in which researchers had observed that major depressive episodes were less prevalent and severe in individuals aged 75 or more compared to younger age groups (Kessler *et al.*, 2010). More recently, the association between depressive symptomatology and age in LLD has been characterised by an inverted U-shape, with a peak between age 70 and 80, followed by a significant decrease in >80 years old patients (Barrenetxea et al., 2022). A possible explanation for such an age-related decrease in depressive symptoms is that only a minority of elderly patients could maintain the same depressive manifestations as adults whereas in most others affective symptoms would be replaced by somatic or cognitive manifestations (Gonda *et al.*, 2009; Skoog, 2011) that are neither included among DSM criteria for major depressive episode nor assessed by common scales for adult depression such as the MADRS. It is worth noting that, although our ≥75 years old subjects were less severely depressed, their levels of suicidal ideation were similar to those detectable in younger individuals. This highlighted the presence of a complex network of suicidal risk factors operating in old age depressive disorders (Fernandez-Rodrigues *et al.*, 2022). In particular, since depression and physical illnesses are both contributors to suicide risk in the elderly population (Obuobi-Donkor *et al*., 2021), the lower levels of depressive symptoms reported in older patients could be counterbalanced by their greater physical burden. The effectiveness of antidepressant medications for LLD is still unclear. If some researchers have found positive results, which led them to recommend antidepressant medications as first-line treatments for elderly depression (Pruckner and Holthoff-Detto, 2017), for others these drugs would only warrant questionable benefits and, therefore, they should be cautiously prescribed to older people (Mallery *et al.*, 2019). The IRL GREY study had already shown a positive response to antidepressant treatments in patients aged >60 (Hall et al., 2015). In our reanalysis, a new finding was that such a response could be maintained in older age. Similarly, a recent meta-analysis has estimated an overall response rate of >50% in elderly depressed patients, reporting no significant age-related variations (Gutsmiedl *et al.*, 2020). However, by controlling for baseline severity, we observed that depressive symptoms had a more clear-cut decrease during antidepressant treatment in ≥75 years old group. Thus, not only antidepressant response was present in older age, but it seemed to be heightened. This possibility has already been discussed commenting on the results of a large population-based study on completed suicide. In that cohort, individuals aged >80 treated with antidepressant medications were less likely to commit suicide than their counterparts without antidepressant therapy, but this difference was not seen in younger age groups (Erlangsen and Conwell, 2014). The impact of antidepressant medications in elderly samples is affected by biological and clinical variables. Biological research has emphasised the role of age-related changes in serotonin function in different brain regions (Goldberg *et al.*, 2004). At the clinical level, it is important how antidepressants are titrated. As a general rule, slow titration should be preferred as it has been associated with better antidepressant response and greater tolerability (Olgiati *et al.*, 2014; Olgiati and Serretti, 2015).

Cognitive impairment is one of the best characterised traits of LLD (Masse *et al.*, 2022) that has been associated with response and attrition during antidepressant treatment, as well as with suicidal behaviour (Cristancho *et al.*, 2018; Jordan *et al.*, 2020; Lin *et al.*, 2021; Pan *et al.*, 2022). The degree of cognitive dysfunction was suggested to be higher in LOD in comparison with the EOD subtype (Ly *et al*., 2021). Before concluding that such differences are real; however, it is necessary to unravel possible confounding factors. For example, Cheng *et al*. (2020) have reported no associations between cognitive function and age of onset in a sample of older adults with remitted depressive disorders. To comment on their negative findings these researchers have argued that in previous studies the relationship between cognitive impairment and LOD could have been biased by active or residual depressive symptoms. We analysed the confounding effect of age. In our sample, there were several cognitive differences between LOD and EOD groups: although most of them were related to chronological age rather than to the age of onset, suggesting they could simply reflect the impact of brain ageing (Diniz *et al.*, 2021), memory dysfunction has emerged as a true feature of LOD. This finding was consistent with prior studies such as Mackin *et al.* (2014), who reported a correlation between memory impairment and LOD in older adults with depression and executive dysfunction and, more recently, a study of healthy older individuals who later developed depression, among whom mild cognitive impairment preceded depression onset (Almdahl *et al.*, 2022). Another characteristic that was associated with LOD in our sample was an increased risk of developing dyskinesias, notwithstanding the assumption of lower antidepressant doses. This could be more easily understood by considering that in older subjects who develop Parkinson’s disease the onset of this disorder is frequently preceded by LOD (Gustafsson *et al*., 2015; Wang et al., 2018; Kazmi *et al*., 2021). Hence, there might be a greater instability of dopamine circuits in some patients who develop depression in late life (Taylor et al., 2022), which would also facilitate the onset of movement disorders.

The most evident weakness of our analysis was the small size of the LOD group. This caused a loss of statistical power and, as a consequence, we cannot exclude that a number of existing differences between LOD and EOD were overlooked. A further weakness was to have assessed depressive symptomatology by means of the MADRS. In fact, this scale has not been specifically developed for geriatric depression and does not cover somatic and other commonly reported depressive symptoms that are common in the elderly population. On the other hand, a strength of this study was the possibility of analysing a broad set of clinical variables thanks to the accurate phenotypic characterisation of this sample.

In conclusion, our results suggest that ageing process might exert a strong pathoplastic effect on depressive disorders which firstly occurred in young adulthood; further research is needed to elucidate the underlying biological mechanisms in order to better tailor LLD treatments according to patients’ ages. On the other hand, specific changes in brain function may occur in old age, thereby facilitating the onset of depression, memory dysfunction, and movement disorders in previously healthy individuals.

## Acknowledgements

We thank the NIMH for having had the possibility of analysing their data on the Incomplete Response in Late Life Depression: Getting to Remission (IRL GREY) sample. We also thank the authors of previous publications in this dataset, and foremost, we thank the patients and their families who accepted to be enrolled in the study. Data and biomaterials were obtained from the limited access datasets distributed from the NIH. The ClinicalTrials.gov identifier is NCT00892047.

### Conflicts of interest

A.S. is or has been a consultant/speaker for Abbott, Abbvie, Angelini, AstraZeneca, Clinical Data, Boehringer, Bristol-Myers Squibb, Eli Lilly, GlaxoSmithKline, Innovapharma, Italfarmaco, Janssen, Lundbeck, Naurex, Pfizer, Polifarma, Sanofi, Servier, and Taliaz. For the remaining author, there are no conflicts of interest.
